# Supplementary figures and images for: Quantification of migrant hoverfly movements (Diptera: Syrphidae) on the West Coast of North America
Source: R Soc Open Sci. 2019 Apr 3;6(4):190153. doi: 10.1098/rsos.190153 (PMC6502382; doi:10.1098/rsos.190153)

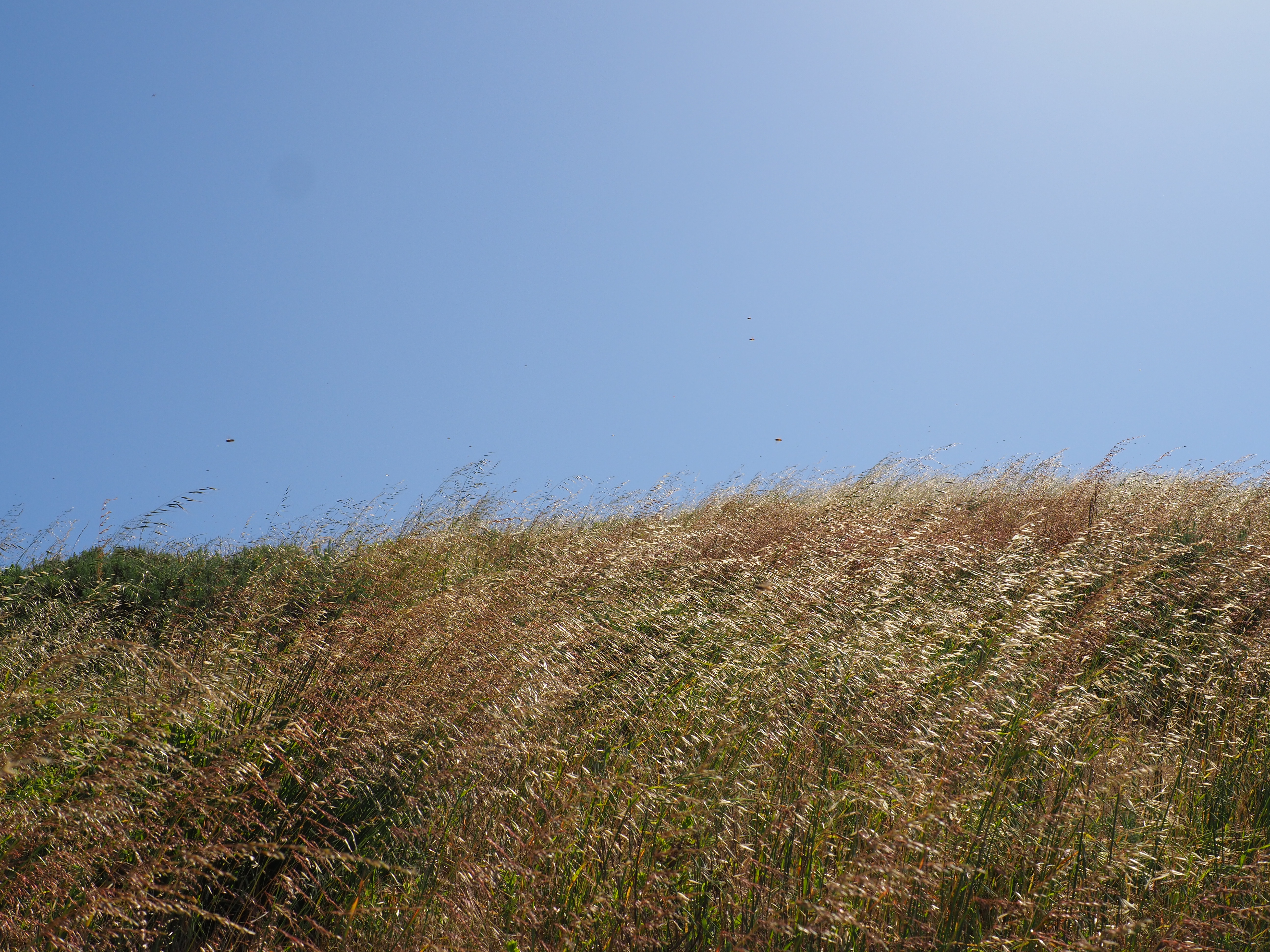

Supplement: High-resolution still image of the hoverfly migration [file rsos190153supp2.jpg]

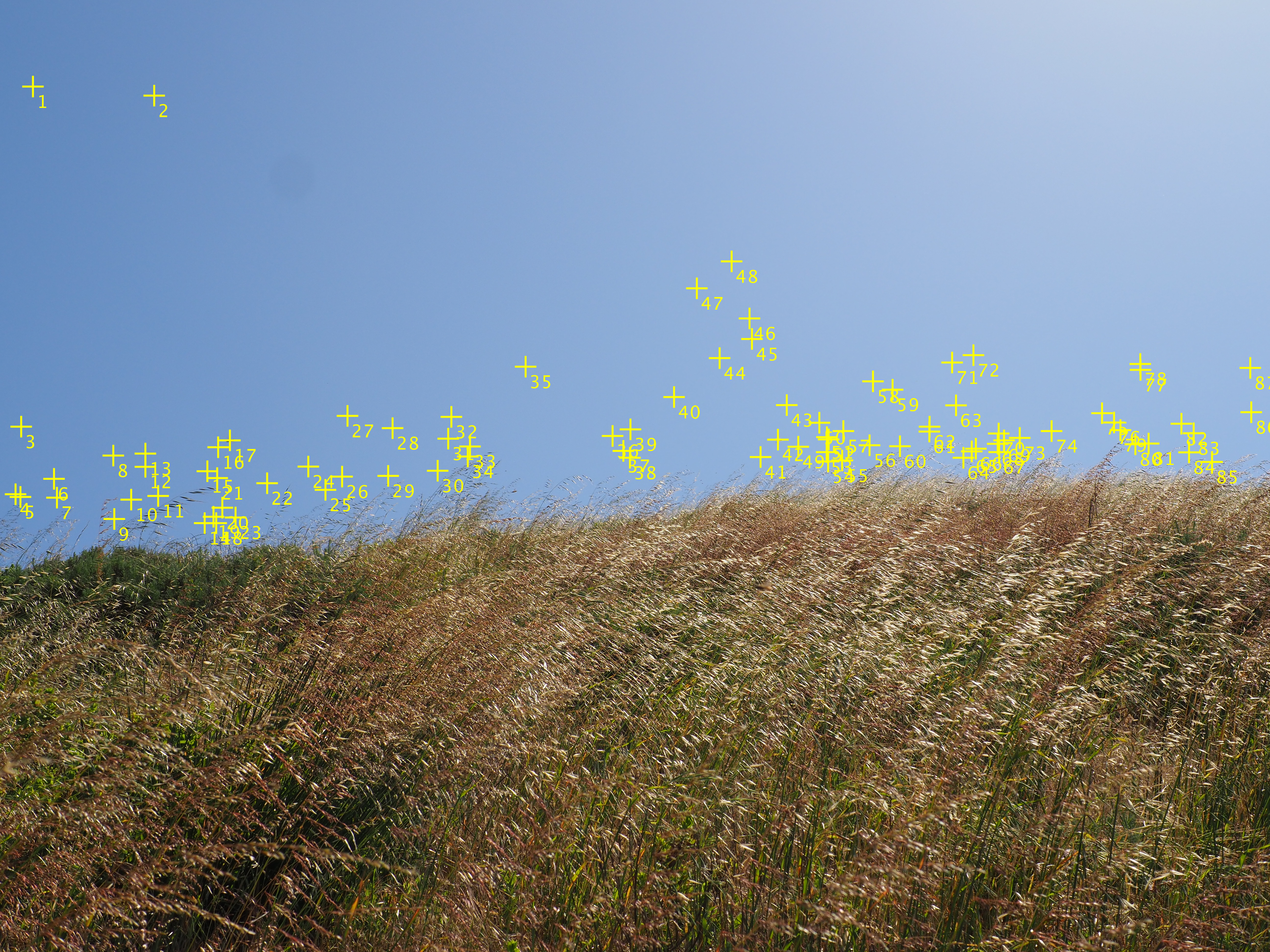

Supplement: Annotated high-resolution still image of the hoverfly migration [file rsos190153supp3.png]
